# Global Medical Device Nomenclature: The Concept for Reducing Device-Related Medical Errors

**DOI:** 10.4103/0975-1483.71637

**Published:** 2010

**Authors:** K Anand, SK Saini, BK Singh, C Veermaram

**Affiliations:** *Department of Pharmaceutics, Faculty of Pharmaceutical Sciences, Jodhpur National University, Jodhpur, Rajasthan*; 1 *Medical Affairs, Johnson and Johnson Limited, Gurgaon, Haryana, India*; 2 *Department of Pharmaceutical Sciences, Kumaun University, Nainital, Uttarakhand, India*

**Keywords:** CEN, coding system, device safety, ISO, medical errors, Unique device identification

## Abstract

In the medical device field, there are a number of players, having quite different responsibilities and levels of understanding of the processes, but all with one common interest, that of ensuring the availability of sound medical devices to the general public. To assist in this very important process, there is a need for a common method for describing and identifying these medical devices in an unambiguous manner. The Global Medical Device Nomenclature (GMDN) now provides, for the first time, an international tool for identifying all medical devices, at the generic level, in a meaningful manner that can be understood by all users. Prior to the GMDN, many nomenclature systems existed, all built upon different structures, and used locally or nationally for special purposes, with unusual approaches. These diverse systems, although often workable in their own right, have had no impact on improving the overall situation of providing a common platform, whereby, medical devices could be correctly identified and the related data safely exchanged between the involved parties. Work by standard organizations such as, CEN (European Committee for Standardization) and ISO (International Organization for Standardization), from 1993 to 1996, resulted in a standard that specified a structure for a new nomenclature, for medical devices. In this article we are trying to explain GMDN as the prime method to reduce medical device errors, and to understand the concept of GMDN, to regulate the medical device throughout the globe. Here we also make an attempt to explain various aspects of the GMDN system, such as, the process of development of the GMDN-CEN report, purpose, benefits, and their structural considerations. In addition, there will be an explanation of the coding system, role of the GMDN agency, and their utilization in the unique device identification (UDI) System. Finally, the current area of focus and vision for the future are also mentioned.

## INTRODUCTION

Within all regulations concerned with medical devices, there are a number of obligations placed upon the manufacturer. In addition, the authorities are faced with the task of regulating the manufacturers and their devices, and there are the people involved in the trade of these devices (e.g., the suppliers), before the devices themselves are brought into use. Finally, of course, there is the myriad of users, who, when the devices initially arrive at the place of intended use, struggle with an important task of correctly identifying and registering these devices in their local databases. This means that there are a number of players, having quite different responsibilities and levels of understanding of the processes, but all with one common interest, that of ensuring the availability of sound medical devices to the general public. To assist in this very important process there is a need for a common method for describing and identifying these medical devices in an unambiguous manner. The GMDN now provides, for the first time, an international tool for identifying all medical devices at the generic level, in a meaningful manner, which can be understood by all users.

Prior to the GMDN, many nomenclature systems existed, all built upon different structures, and used locally or nationally for diverse purposes and with unusual approaches. These diverse systems were CNMD, EDMA, ISO 9999, JFMDA, NKKN, UMDNS, and so on, and furthermore these were used to develop global medical device nomenclature systems. Although often workable in their own right, they had no impact on improving the overall situation of providing a common platform whereby medical devices could be correctly identified and the related data safely exchanged between the involved parties. The advent of the European directives, initiated a new era, where national and indeed international bodies were given the opportunity to cooperate and harmonize their efforts in achieving the one thing that they all needed, namely, a standardized method of identifying the products placed in the global market.

Work by standard organizations like CEN and ISO, from 1993 to 1996, resulted in a standard that specified a structure for a new nomenclature for medical devices. This standard, now revised by ISO, is published as the ISO 15225 Nomenclature – Medical device nomenclature data structure. Following this, a project was set up in 1997, by the CEN, with financial support from the European Commission (EC). The aim of the project was to create a comprehensive nomenclature for all medical devices, suitable for use by all interested parties globally.[1]

To facilitate the rapid production of the GMDN, six existing nomenclatures of particular standing were adopted. These covered a wide range of terms defining medical devices and healthcare products that combined, gave a total of 13,500 terms.

The six chosen nomenclatures were:

- Classification Names for Medical Devices (CNMD) and *in vitro* Diagnostic Products. Developed by the Food and Drug Administration (FDA), USA.
- European Diagnostic Manufacturers Association (EDMA) *in vitro* diagnostic product classification. Used in Europe.
- ISO 9999 Technical Aids for Disabled Persons Classification. International use.
- Japanese Medical Device Nomenclature (JFMDA). Used in Japan.
- Norsk Klassifisering Koding and Nomenklatur (NKKN), Norwegian Nomenclature.
- Universal Medical Device Nomenclature System (UMDNS). Developed by ECRI, USA.

On the 1^st^ November, 2001, the Global Medical Device Nomenclature (GMDN) was published as a CEN Report CR 14230 and as ISO. TS 20225. The first public release on CD-ROM as GMDN version 2002.1 was in November 2002.[1]

### Development of GMDN-CEN Report CR 14230

Medical Device experts from around the world (manufacturers, healthcare authorities, and regulators) compiled the initial GMDN nomenclature based on the standard ISO 15225. The study was mandated by the European Commission in order to provide the necessary tool to carry out many of the obligations following the implementation of the Medical Devices Directives, and also, to meet similar needs at the Global level, as identified in the Global harmonization activities of GHTF by its members, notably USA (FDA), Canada, European member states, Japan, Australia, and now by many other countries / regions.[2]

In 1991, the first international workshop on harmonization of Medical Devices Nomenclature was held among EU, EFTA, Canada, and USA. However, there was no fruitful outcome of this meeting. Consequently in 1993, as requested by the Commission, CEN established a standard to define a structural basis for a device nomenclature. Subsequently symbols, coding, and proposed nomenclature were developed by USA workgroups in 1994 and 1995; there was a CEN recommendation for an interim nomenclature system.[3] Adding to these, in 1996, a project proposal for a device nomenclature system was submitted and it was positively accepted by the European nomenclature standard, through the Vienna agreement.[2] All this resulted in one single harmonized standard. There was also a license agreement between ECRI and CEN for the use of their UMDNS (Universal Medical Device Nomenclature System) as one component of the development process. From 1997 to 1999, the GMDN Project was undertaken, based on the structural standard. It defined the general structure of the nomenclature and provided the required understanding of field lengths, data relationships, and so on. The project involved some 70 medical device experts from 16 countries, a Project Council of 10 members, an Expert Advisory team of six members, and a secretarial / support team of six members. Finally in 2001, the GMDN Nomenclature as the CEN Technical Report CR 14230, was released, which was identical to the ISO Technical Specification ISO TS 20225.[2] There was also the establishment of the GMDN Maintenance Agency Policy Group, which was responsible for the future management of the GMDN as also to develop and distribute the electronic version of the GMDN and all future versions of the data file. BSI (British Standards Institution) as delegated by CEN has recognized the GMDN Agency to be the formal body for the ongoing management and control of the GMDN on a global basis. The GMDN Agency has therefore exercised, and will continue to exercise, the sole rights to develop and distribute all versions of the GMDN and its associated data, terminology, and supporting databases.[3]

### Purpose of global medical device nomenclature

The foremost purpose of the GMDN is to provide a single, global, nomenclature system by which the authorities can regulate medical devices; this is also impacting upon the healthcare providers, who are the mainstay users of medical devices, the medical device manufactures, suppliers, conformity assessment bodies, and other affiliated parties, so that there is only one single system that provides generic product descriptors to support patient safety.[1] The GMDN code represents the generic descriptor (this being the term name along with its definition) in order to internationally standardize device identification, for reasons of safe data exchange between competent authorities and others, exchange of post-market vigilance information, research, medical record keeping, e-commerce, and inventory purposes.[4]

### Benefits of global medical device nomenclature

Internationally, it provides a common terminology, enabling global regulatory partners to efficiently communicate and share medical device details. If we talk about the national benefits, it provides a common terminology, enabling the federal entities and the industry to efficiently communicate and share medical device data. DHHS promotes the work on the GMDN and consolidates with UMDNS, which anticipates a federal government standard in a few years. As far as the Regulatory agency is concerned, the GMDN provides guidelines for the appropriate and consistent naming of the device groups and the creation of descriptive definitions that are device-group specific. Specifically, their utilization by regulators will help to improve nomenclature practices, cross-generational consistency, and data management, for post-market activities.[4]

### Global medical device nomenclature structure

**Figure 1:**
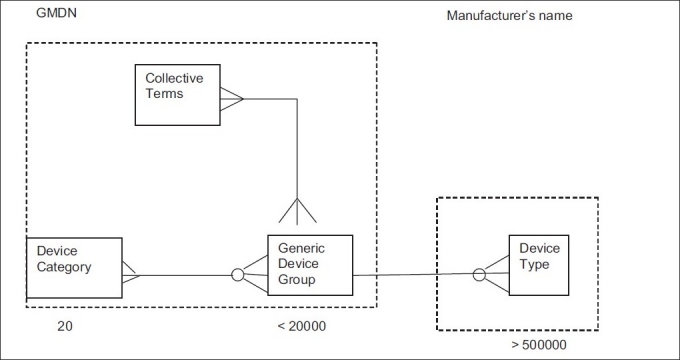
General organization of GMDN data

The general structure of the GMDN is regulated by requirements stipulated in the standard ISO 15225 Nomenclature – Medical device nomenclature data structure. Figure 1 shows the basic organization of the GMDN data. The data is defined by three levels, associated with an external fourth level, each level containing data that differs in the degree of specificity.[5]

### Device category

The device category is the broadest level of the GMDN data. It divides the entire medical device product market into the highest-level groups based on device application, technology, or other common characteristics. The standard allocates codes for possibly 20 categories; there are currently 16 established device categories.[6] These are:

**Table:** 

Code	Code
01	Active implantable devices
02	Anesthetic and respiratory devices
03	Dental devices
04	Electromechanical medical devices
05	Hospital hardware
06	*In vitro* diagnostic devices
07	Non-active implantable devices
08	Ophthalmic and optical devices
09	Reusable devices
10	Single use devices
11	Assistive products for persons with disability
12	Diagnostic and therapeutic radiation devices
13	Complementary therapy devices
14	Biological-derived devices
15	Healthcare facility products and adaptations
16	Laboratory equipment
17	Reserved
18	Reserved
19	Reserved
20	Reserved

### Generic device group

The generic device group is the most specific level at which products are aggregated, based on common technology or intended use. There are four different types of GMDN terms associated with the generic device groups. These terms, with their alpha identifiers, include the:

**Table:** 

Preferred term	P
Template term	T
Synonym term	S
Multiple-linked synonym term	MS

There are also terms within the main database called ‘Equivalent terms’. These are simply nonactive terms from the original six source nomenclatures used to develop the GMDN and can be equal to a P, T, S, or MS term within the database. They are identified with the alpha identifier E.

Using the GMDN search engine, or using some of the view possibilities provided by the database, E terms can be found and viewed. The GMDN user is not to be concerned with equivalent terms, as they are not active terms, they have no GMDN code allocated, and cannot be used by a user for any practical application.

### Preferred terms

Preferred terms with their unique five-digit codes are the only terms available for product identification. The preferred term is the optimal name selected to represent a group of devices (a collection of device types) that have the same or similar intended use or commonality of technology allowing them to be grouped in a generic manner, typically without reflecting the specialized characteristics, such as, brand or trade names.

Each preferred term has an associated definition that describes the most prominent characteristic of the device types in the group [typically includes a physical description and an intended use(s)]. It is the definition that determines the scope of the preferred term and code. The preferred term type identifier is (P).

### Template terms

The template term is a general device name added to the nomenclature when multiple preferred terms have identical character strings forming a base concept, and functions as a header term to create a simple hierarchy for lexically-related preferred terms.

Each template term has an associated definition that is inclusive of all subordinated preferred terms. The template term type identifier is (T). The template term is a navigational tool only and must not be used for product identification purposes.

If a template term code has been used to identify any products in the past, this code has been wrongly applied and is not valid. A GMDN P term and code that is applicable to the product must be selected and assigned instead. If no current GMDN P terms are applicable, an application for the creation of a new term or modification of an existing P term must be submitted.[5]

### Synonym terms

The synonym term is the common use or a familiar name used in the nomenclature. It is an alternative entry point in the nomenclature, used to locate the preferred term or template term to which it is linked. It may or may not actually be synonymous with the term to which it is linked. The synonym term type identifier is (S). Selecting a synonym term in the GMDN database will automatically produce its linked preferred or template term. The synonym term is a navigational tool only and must not be used for product identification purposes.

### Multiple-linked synonym term

The multiple-linked synonym term is a medical device name, often from one of the GMDN source nomenclatures, that is typically of a higher-order, and is therefore linked to more than one preferred term and / or template term. The multiple-linked synonym term type identifier is (MS). This multiple-linked synonym term is a navigational tool only and must not be used for product identification purposes.

### Collective terms

Collective terms are used to aggregate medical device groups that have common features and are identified in the GMDN. Although the GMDN has been designed and developed for regulatory data exchange in areas such as vigilance reporting and tracking of medical device safety, there is a need for a set of terms that are more refined than the GMDN category terms, yet broader than the GMDN generic device group terms, to be used in the application of the medical device directives; the use of collective terms satisfies this requirement.[5]

Collective terms are intended to be used for a whole range of subject matters, for example:

- To illustrate the scope of certificates issued by Notified Bodies when assessing which groups, families or types of medical devices are covered within a manufacturer’s quality system
- To be used to identify the range of skills and general technological abilities for which a Notified Body has been approved, and is so appointed by the relevant Competent Authority
- For exchanges of information between Competent Authorities when general information on individual manufacturers capabilities is notified for inclusion in the European Database for Medical Devices (EUDAMED)

There were several means identified to aggregate the generic device group terms of the GMDN using collective terms. These were:

- Devices covered by the application of common technology
- Devices manufactured using similar manufacturing procedures and common technical features
- Devices manufactured for the application of similar Medical Procedures
- Devices manufactured using common materials requiring special skills
- Devices developed to meet specific risk-associated considerations

For the ease of electronic transmission of data and code recognition, each collective term is assigned a four-digit incremental code, with the prefix ‘CT’ (Collective Term).

### Coding system for GMDN

All terms in the GMDN are assigned a unique code. This provides security in cases of misunderstandings, language barriers, or discrepancies in the data systems. The code is an incremental, sequential cardinal number comprising of five digits starting from 10000. The codes in themselves are not created with an integral hierarchical structure and are simply unique numbers. The codes are the carriers of the information to which they are linked and should always be used and referred to in any reference to the GMDN or data transaction. There are only three categories of ranges that are used in the coding system for GMDN.[2]

They are:

- Codes in the range of 1 – 9999
- Codes in the range of 10000 – 30000
- Codes above the range of 30000

**Codes in the range of 1 – 9999** are not represented in the GMDN. These, as stated in the standard, are exclusively reserved for assignment by any end user and may be used as desired in any user’s local data system. It is important for readers to understand that this range of codes should not be used for any kind of official purpose, for example, as temporary codes, as national translated synonyms, or where the data is exchanged between users outside of the local data system. This will lead to ambiguity.

**Codes in the range of 10000 – 30000** are represented in the GMDN and have been reserved exclusively to represent the original code used by the ECRI organization for their UMDNS terms that have been adopted for use in the GMDN. This will provide the GMDN user with automatic mapping from the ECRI code representing the UMDNS term to the identical GMDN code used at present to represent the GMDN term. This has been done to assist in the transition for users who have previously used the ECRI UMDNS.

**Codes above the range of 30000** are all GMDN, created for GMDN terms.[2]

### Role of GMDN agency/governance

The need for a Maintenance Agency was identified and the structure was approved within the CEN. Therefore, in order to manage the GMDN, a maintenance agency was set up to form the necessary legal entity. This non-profit company, the GMDN Agency, acting as the Maintenance Agency Secretariat (MAS), functioned as the hub in the running and maintenance of the GMDN. It also provided services and information for access to the GMDN data through the Internet site or other means. To ensure continuing permanency of the GMDN, revenues could be generated through the licensing or sale of GMDN Agency products and services, or by direct funding, allocated by relevant global regulatory bodies or other parties.[5]

Services provided by the GMDN agency are:

- Access to the GMDN data file and codes using the Internet site through a license agreement and / or by direct credit card purchase
- A link from the GMDN database to the user’s in-house data system through a license agreement and a custom-made software link
- Application form for new terms or modification of existing terms / definitions for the identification of the user’s product
- Access to GMDN terminology and information
- Guidance on how to use the GMDN
- Access to Collective terms
- GMDN translational software tools

A Maintenance Agency Policy Group (MAPG), now an entity of the GMDN Agency, ensures a globally recognized representation that consists of delegates from authorities and the industry, as well as other interested parties. Based on the original Technical Report a vastly developed and enhanced GMDN is now available through the GMDN Agency.

The main role of the GMDN Agency is in accordance with its Memorandum of Association as follows:[5]

- To carry out the functions of a Maintenance Agency to develop and maintain the ‘Global Medical Device Nomenclature’ for medical devices
- To meet the needs identified by the European Commission when developing the European Directives on Medical Devices, to establish a nomenclature for medical device generic descriptors that will meet a global need for identification purposes. This identification will facilitate exchange of regulatory data and assist in the identification of medical devices for commercial purposes
- To ensure that the GMDN meets the needs of all national authorities, the industry, and other users globally, as the primary reference and working generic nomenclature for the exchange of regulatory and commercial information
- To ensure that the GMDN is constructed with reference to European and International standards, indicating the structure of such a medical device nomenclature (e.g., EN ISO 15225)
- To be responsible for adding, amending, and archiving terms and definitions for medical devices and to assign codes as required, to provide easy identification. Such codes will be consistent so that all translated versions of the nomenclature will carry an identical code for each generic or other term as specified in the GMDN
- To liaise with standards bodies (e.g., CEN, CENELEC, ISO, IEC), to be aware of the current standards on medical device nomenclature (including any additional levels of identification, e.g., collective terms, identification of particular attributes, and links to other nomenclatures as appropriate)
- To coordinate and link with appropriate organizations for the translation of GMDN into other languages

### Utilization of GMDN in unique device identification

For the sake of patient safety, in the era of global economy, it is desirable to address tracking and tracing of medical devices at a global level. One reliable way to achieve the track and trace of medical devices is to develop a Unique Device Identifier (UDI). The primary aim of a UDI mechanism is to increase patient safety. It will also improve the work of market surveillance authorities in case of field safety corrective actions, for instance, the fight against counterfeiting. In addition, the development of an international approach will make the trade of medical devices more secure for all the stakeholders (health authorities, hospitals, manufacturers, distributors, etc.).[4]

This is why the introduction of GMDN in a UDI system appears to many regional regulatory authorities and to the industry at large, as an effective tool to protect public health more efficiently. It is mainly for reasons of patient safety that all the actors of the sector advice development of UDI for medical devices. Therefore, common worldwide UDI requirements would offer significant benefits to the manufacturer, user and / or patient, and the Regulatory Authorities. In addition, eliminating or reducing differences between jurisdictions decreases the cost of gaining regulatory compliance. Second, while developing a UDI system, one important consideration which should be taken into account is the risk associated with the device. The UDI mechanism should be implemented stepwise according to the risk of the device — starting with the highest risk first (e.g., implants) and staggering the implementation, with the lowest risk class (e.g., most disposables) implemented last. Furthermore, the introduction of the UDI should allow sufficient implementation timeframes, to allow the manufacturers to comply with the requirements. Finally, in order to achieve all the positive elements of a UDI mechanism, the use of the UDI should be promoted among all stakeholders, including regulatory agencies, medical device manufacturers, distributors, hospitals, and medical professionals.

A UDI for medical devices will consist of a unique identification code using a globally accepted standard format like the GMDN. In order to accommodate most methods of labeling, marking, and identifying products, the UDI should be technology neutral, that is, it should not be restricted to a particular method of Automatic Identification and Data Capture (AIDC).[4] The UDI database shall allow the use of an existing globally accepted data exchange process to harmonize the exchange of device information, for safety purposes. It should utilize a globally accepted nomenclature such as the Global Medical Device Nomenclature (GMDN).

Current areas of focus with a vision for the future

- There should be a continued efforts to consolidate UMDNS and GMDN
- Continue to prepare for harmonized regulatory utilization of GMDN by developing requirements for the integration of GMDN data into device registration as well as approval applications
- We should continue working on the system for the unique identification of products, to potentially be associated with the GMDN
- In future a high-quality medical device terminology should be the standard that promotes good data management, which should be associated with unique device identification[4]

## SUMMARY

Global Medical Device Nomenclature is an internationally recognized system of names, definitions, and codes used to accurately identify and discretely describe generic medical device groups primarily for product certification / registration, vigilance reporting, and product recall. The foremost purpose of the GMDN is to provide a single, global, nomenclature system, by which the authorities can regulate medical devices; this is also impacting upon the healthcare providers, who are the mainstay users of medical devices, the medical device manufacturers, suppliers, conformity assessment bodies, and other affiliated parties, so that there is only one single system that provides the generic product descriptors, to support patient safety. Specifically, their utilization by all stakeholders will help to improve nomenclature practices, cross-generational consistency, and data management, for post-market activities. To summarize, we can say that after adopting the GMDN concept there may be,

- Common terminology for efficient data sharing and communication
- Improved nomenclature practices and device group identification
- Potential for a system of unique device identification that links device-group information with device-type information
- Essentially there will be improved data management for post-market activities, which will reduce medical device errors

## References

[1] Gmdnagency.com. Global Medical Device Nomenclature; User Guide. http://www.gmdnagency.com/.

[2] Gmdnagency.com. Global Medical Device Nomenclature. http://www.gmdnagency.com/?id=userinfo.

[3] Ghtf.org. Global Harmonization Task Force; Generating the GMDN Process and Development. http//www.ghtf.org/meetings/conferences/8thconference/…/Dillard.ppt.

[4] Ahrq. gov. Agency for Health Research and quality; Brockton Hefflin; Center for Devices and Radiological Health; Food and Drug Administration. http//www.healthit.ahrq.gov/portal/server.pt?open=18.ppt.

[5] Gmdnagency.com. Global Medical Device Nomenclature. http://www.gmdnagency.com/?id=userinfo.

[6] Ahwp. info. Asian Harmonization Working Party; Report from AHWP STG Nomenclature. http://www.ahwp.info/ClientFolder/AHWP/Library/Tree/4_Technical_Committee/3_TC_Minutes/The_9th_AHWP_Technical_Committee_Meeting/14_Medical_Device_Nomenclautre.pdf.

